# Clinical Profile and Limitations in the Management of HBV Patients Attending Clinic at a District Hospital in Ghana

**DOI:** 10.1155/2023/4424718

**Published:** 2023-01-04

**Authors:** Amoako Duah, Yvonne A. Nartey

**Affiliations:** ^1^Department of Medicine, University of Ghana Medical Centre LTD, Accra, Ghana; ^2^Department of Medicine, Cape Coast Teaching Hospital, Cape Coast, Ghana; ^3^Department of Internal Medicine, School of Medical Sciences, University of Cape Coast, Ghana

## Abstract

**Background:**

Chronic hepatitis B (CHB) is estimated to cause between 500,000 and 1.2 million deaths worldwide every year through cirrhosis and hepatocellular carcinoma (HCC). Liver cirrhosis and HCC are the commonest liver diseases causing death in Ghana. The most critical problem in the management of CHB in sub-Saharan Africa is the high cost of investigations and antiviral drugs. There is scanty information concerning newly diagnosed CHB patients and their management challenges in Ghana. This study sought to determine the clinical characteristics and management challenges of CHB patients in Ghana. *Methodology*. A prospective cohort study was conducted involving newly diagnosed CHB patients being managed at St. Dominic Hospital. Patient demographic and clinical features were abstracted using a standardized questionnaire. The proportion of patients able to undertake investigations and treatment were determined, and the limitations to standard management were recorded. The performance of APRI score in the diagnosis of cirrhosis was also investigated.

**Results:**

Of the 334 patients with newly diagnosed CHB, the median age at diagnosis was 35 (IQR 28–44) years. Less than a quarter (22.2%) were able to undertake viral load testing and 23.4% were eligible for treatment. Of those who were eligible for treatment, only 42.3% were able to initiate treatment. Almost a third of cases (32.1%) reported late with liver-related complications. The sensitivity of APRI score with cut-off value of 2 in the diagnosis of liver cirrhosis was 70.2% and specificity was 97.9%.

**Conclusion:**

A high proportion of newly diagnosed CHB patients presented late and with liver-related complications. Majority were not able to afford viral load testing and antiviral medication. Screening of hepatitis B among the general population and inclusion of CHB management in the National Health Insurance Scheme should be encouraged.

## 1. Introduction

Hepatitis B virus (HBV) is a major contributor to the burden of infectious diseases worldwide. Approximately 250 million of the world population are chronic carriers of HBV [1]. In highly endemic regions, including Africa, the Amazon Basin, and Central and Southeast Asia, the prevalence of HBV is estimated to be up to 8%, reaching 10–15% in some countries [2]. The prevalence of HBV in Ghana is estimated to be 12.3% [3]. HBV is projected to cause between 500,000 and 1.2 million deaths every year through liver failure, cirrhosis, and hepatocellular carcinoma (HCC) [4]. Hepatitis B is the cause for almost half of all cirrhosis diagnoses and 80% of HCC worldwide [4]. Liver cirrhosis is the commonest liver disease causing death at the Korle-Bu Teaching Hospital in Ghana, and liver cancer is the leading cause of cancer death in the country [5]. HBV is the principal cause of both liver cirrhosis and HCC per previous studies conducted in Ghana. Viral suppression or control of viral replication has been established to significantly decrease the risk of cirrhosis and its complications, including HCC [6], and even to induce regression of fibrosis and cirrhosis [7]. Treatment for hepatitis B has been significantly enhanced by the advent of potent nucleos(t)ide analogs (tenofovir and entecavir) with high barriers to resistance, which are paving the way to widely reduce the morbidity and mortality of the disease in wealthy regions. By contrast, in developing countries with high endemicity, access to hepatitis antiviral therapy is still very limited [8]. The World Health Organization (WHO) issued treatment guidelines for hepatitis B in 2015 [9]. They recommend prioritizing antiviral treatment for those with established cirrhosis (based on noninvasive measures of fibrosis), clinical evidence of decompensated liver disease, or evidence of active disease as indicated by abnormal transaminases and a high HBV DNA load (above 20 000 IU/mL) [9]. Treatment guidelines for HBV in Ghana recommend that treatment be initiated in the following categories of patients: all patients with chronic active HBV infection (HBeAg-negative chronic HBV (ALT > 2 × ULN and HBV DNA > 2000 IU/mL) or HBeAg-positive chronic HBV (ALT > 2 × ULN and HBV DNA > 20,000 IU/mL) and all HBV-related cirrhosis or advanced fibrosis (APRI score > 2) patients with detectable viraemia [10].

Chronic hepatitis B (CHB) is an expensive disease to manage. Evaluation of a patient involves collecting a thorough history, detailed physical examination, laboratory tests, and imaging studies. These tests include serological viral markers, biochemical tests to assess hepatic injury, hormone assays, hematological tests, imaging studies, molecular biological studies (HBV DNA viral load assessment), and liver biopsy. These tests must be performed during initial assessment and treatment monitoring and at posttreatment follow-up. Many laboratories in sub-Saharan Africa including some teaching and specialist hospitals in Ghana can only conduct HBsAg screening, serological biomarkers, hematological, and liver function tests but not viral load [8]. The most critical problem in the management of CHB in Africa including Ghana is the high cost of laboratory tests and drugs. One of the barriers identified in this country to HBV treatment is the high cost; however, this was based on qualitative studies [11, 12]. Anecdotal evidence suggests that drugs for treatment may not be widely available, especially in district hospitals in Ghana and even if available may be too expensive for patients to buy. There is little published literature from Ghana concerning the clinical profile of patients with CHB and the challenges in their management. This study therefore seeks to document the clinical profile of patients with CHB and quantitatively determine the management challenges in a district hospital in Ghana. This will help policymakers develop specific protocols for investigating and managing patients with HBV in Ghana. This study will also give an idea of the proportion of chronic HBV patients in this country who will require treatment and possibly come out with ideas of financing their treatment based on the challenges that will be identified. Studies on the clinical profile and challenges in managing HBV in sub-Saharan Africa especially in Ghana are scanty; therefore, this study will add to the body of evidence from other previous studies and help in managing these patients better.

## 2. Methodology

### 2.1. Study Design

This was a prospective hospital-based study.

### 2.2. Study Site

The study was conducted at the St. Dominic Catholic Hospital between 1 January 2018 and 24 June 2020. St. Dominic Hospital is a 356-bed capacity secondary referral center and district hospital located in Akwatia in the Eastern Region of Ghana.

### 2.3. Study Population

Newly diagnosed chronic HBV patients referred to the outpatient gastroenterology clinic of St. Dominic Hospital were enrolled using consecutive sampling once informed consent was obtained. Some of the patients were referred to the clinic mainly because of evaluation of HBV infection which were picked up incidentally through screening at antennal clinic or community level or screening during blood donation. Some were also diagnosed when they presented with clinical features suggestive of liver cirrhosis or HCC to the clinic.

### 2.4. Inclusion Criteria

1. Newly diagnosed chronic HBV patients
2. Patients who provided informed consent

### 2.5. Exclusion Criteria

1. Chronic HBV patients already on antiviral medications or being monitored at clinic

### 2.6. Sample Size

The sample size was determined using Cochran's formula for sample size calculation. With an estimated prevalence of 12.5% for HBV in Ghana (12), a *Z*-score at 95% confidence level (1.96), and a level of significance of 0.05, the minimum sample size was calculated to be 166. Due to the high attrition rate for prospective studies, 334 participants were consecutively recruited after meeting the study criteria and giving informed consent.

### 2.7. Data Collection

Demographic data and clinical presentation of the patients were obtained using a standardized data collection form. Diagnostic investigations, which were necessary for all newly diagnosed cases, were requested for all patients at the first clinic visit and results recorded. These included hepatitis B serology (hepatitis B s-antigen, hepatitis B e-antigen, HBeAg; hepatitis B e-antibody, HBeAb; hepatitis B core IgG, HBcIgG; hepatitis B-core IgM, HBcIgM); serum hepatitis B virus DNA (HBV DNA); HIV and hepatitis C serology; liver biochemistry and function tests; full blood count (FBC); alpha fetoprotein (AFP); and abdominal ultrasound scan. As a standard practice in many hospitals in Ghana, patients were required to undertake these investigations using their own financial resources. Additionally, patients who fulfilled the treatment criteria were given the options of drugs available, which were also to be paid for out-of-pocket. Patients were then reviewed after 6 months to determine the number who had been able to undertake the necessary investigations or purchase medication and the number who had not. Those not able to come to the hospital physically for review were contacted by phone either directly or through their next of kin.

### 2.8. Ethical Approval and Informed Consent

This study was conducted in accordance with the Helsinki Declaration on Human Experimentation, Sixth Revision (October 2008). Institutional Ethical and Review Committee of the St. Dominic Hospital gave formal approval for this study. The nature of the study was explained to the participants, and those who agreed to participate were asked to sign an informed consent form. For patients with hepatic encephalopathy, written consent was obtained from caregivers.

### 2.9. Statistical Analysis

Patient demographic information such as age and sex was described using mean with standard deviation or median with interquartile range. For categorical variables, frequencies (percentages) were reported. The chi-square test was used to compare differences between groups for categorical variables, and the analysis of variance (ANOVA) and the Kruskal-Wallis test were used to examine differences between groups for continuous variables. Multinomial logistic regression was used to identify predictors of liver cirrhosis and HCC (base outcome asymptomatic HBV infection), after adjusting for confounders including age (continuous) and sex (male and female). To assess the performance of the APRI score, sensitivity, specificity, and positive and negative predictive values were calculated, with the optimal APRI cut-off for determining cirrhosis identified using the Youden index. All tests were two-sided and a *p* value of less than 0.05 was considered statistically significant. Data analysis was performed using Stata, version 17, StataCorp software.

## 3. Results

### 3.1. Demographic Characteristics and Clinical State at Presentation

**Figure 1:**
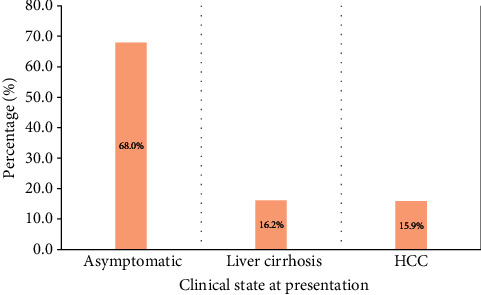
Clinical state at presentation of newly diagnosed chronic HBV cases.

Overall, 334 newly diagnosed chronic hepatitis B patients were recruited during the study period. Of these, 227 (68.0%) presented with asymptomatic CHB, while 15.9% were diagnosed with HCC and 16.2% with liver cirrhosis (Figure 1). The female to male ratio for chronic HBV cases was 2 : 1; however, the opposite trend was seen in liver cirrhosis cases, with a higher male to female ratio of 2 : 1. The majority (86.8%) of HCC cases were male, with a male to female ratio of 7 : 1. The overall median age at diagnosis was 35 years (IQR 28-34). The median age for those with chronic HBV without cirrhosis or HCC was 32 years (IQR 27–39), 45 years (IQR 37–55) for those complicated with liver cirrhosis, and 39 years (IQR 29–46) for those with HCC, with significant difference in ages at the time of diagnosis (*p* = 0.001) (Table 1).

When laboratory results were reviewed, the median APRI score of liver cirrhosis cases was 3.07 and was in line with the recommended cut-off of >2 for the diagnosis of cirrhosis. The median ALT level was higher in cirrhosis (69.0 U/L) and HCC patients (68.9 U/L) compared with chronic HBV patients (*p* = 0.001). There was no statistically significant difference in the HBeAg status between chronic HBV, cirrhosis, and HCC patients (*p* = 0.28). Out of 175 HBeAg-negative patients for whom liver biochemistry results were available, 40 (22.9%) had chronic hepatitis (i.e., elevated ALT level above 40 U/L) and 135 (77.1%) had chronic HBV infection without hepatitis. For those who were HBeAg positive, a higher proportion (57.1%) had chronic hepatitis (Table 2).

### 3.2. Proportion of Patients Able to Undertake Diagnostic Investigations

**Figure 2:**
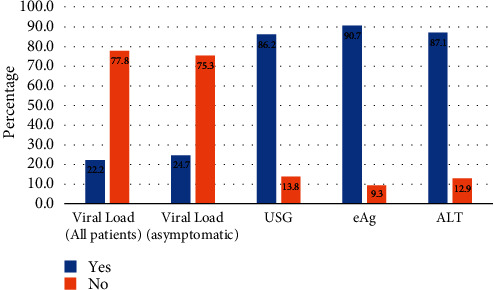
Proportion of patients who were able to undertake diagnostic investigations.

**Figure 3:**
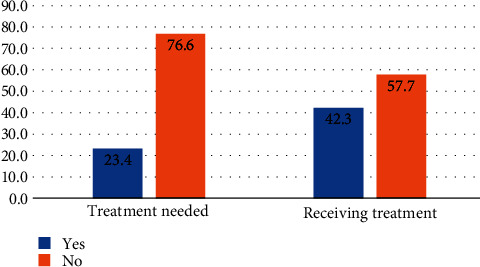
Proportion of patients (a) meeting treatment criteria and (b) able to initiate treatment.

Out of the 334 patients recruited, only 74 (22.2%) were able to undertake HBV DNA testing (Figure 2). For patients with asymptomatic chronic HBV (*n* = 227), for whom the absence of cirrhosis or HCC meant that HBV DNA was essential to determine treatment eligibility (barring any other indications), only 56 (24.7%) were able to pay for and undertake viral load testing. The majority of patients were able to pay for and undertake HBeAg, ALT, and abdominal USG for their clinical workup. Based on the APRI score, viral load (where available), ALT level, and clinical presentation, 78 (23.4%) fulfilled the treatment eligibility criteria, and of these 78, only 33 (42.3%) were able to initiate treatment within 6 months of diagnosis (Figure 3).

### 3.3. Profile of Pregnant Women

Eight-one (81) out of 334 patients were pregnant women, all of whom were asymptomatic. Out of the 81 pregnant women, only 9 (11.1%) were able to pay for viral load testing, and 55 (68.0%) undertook a hepatitis B profile (Table 3). HBeAg was positive in 1 patient (1.8%).

### 3.4. Predictors of Cirrhosis and HCC

In the logistic regression analysis, independent predictors of liver cirrhosis were age (OR: 1.13; CI: 1.07–1.19; *p* = 0.001) and APRI score (CI: 1.59–3.48; *p* = 0.001). Only age (OR: 1.24; CI: 1.06–1.47) was an independent predictor of HCC (Table 4).

### 3.5. Performance of APRI in the Diagnosis of Cirrhosis

The sensitivity of APRI score with a cut-off value of 2 in the diagnosis of liver cirrhosis was 70.2% and the specificity was 97.9% (Table 5). This suggests that the APRI score may miss some patients with cirrhosis who require treatment. There was improved sensitivity without much loss of specificity, 80.6% and 95.5%, respectively, when the APRI cut-off value was revised to 1.1, based on analysis using the Youden index.

## 4. Discussion

This study is aimed at determining the clinical profile and limitations in managing HBV patients attending a clinic at a district hospital in Ghana. The World Health Organization adopted the Global Health Sector Strategy on Viral Hepatitis in 2016 with the goal of eradicating viral hepatitis as a public health threat by 2030 by reaching targets that principally aim to reduce new hepatitis infections by 90%, treat 80% of viral hepatitis patients, and reduce death by 65% [13]. HBV-infected patients are at increased risk of death from liver cirrhosis, hepatocellular carcinoma (HCC), and liver failure. In Ghana, HBV is the leading cause of liver cirrhosis and hepatocellular carcinoma, and this condition normally afflicts a younger population compared to Western counterparts, leading to mortality and its consequent economic hardship for families affected [12–15]. Although the majority of patients in this study presented with asymptomatic clinical features, a relatively high number (32.1%) reported late with liver-related complications (liver cirrhosis and hepatocellular carcinoma). This is similar to a proportion of 33.3% reported by Archampong and Nkrumah [16], in a study conducted at a teaching hospital in Ghana, but is lower than a Spanish study by Picchio et al. [17] in which roughly 15% of patients presented at a late stage. Other studies have reported worrying trends of patients with viral hepatitis reporting late for care [18, 19]. Late presentation reflects a failure of the health care system to diagnose infected patients early enough to prevent complications of HBV. Expansion of screening, training to increase the knowledge of primary health care workers regarding HBV, linkage of positive individuals to care, raising awareness about effective and safe drugs for treatment, and ensuring that the National Health Insurance Scheme (NHIS) takes care of the cost of treatment may reduce late presentation of patients with HBV in Ghana.

Although female participants in this study were more than males, more males presented with liver cirrhosis and HCC than females. This is comparable to other studies conducted in this country and other published studies from other countries [12, 14–16]. Possible reasons for gender inequality of chronic liver disease include gender-specific lifestyle and involvement of activities that expose them to risk factors of chronic liver disease such as excessive alcohol consumption, smoking, intravenous drug abuse, poor dieting, and HBV or HCV. Sex hormones have also been found to play a role as postmenopausal women tend to be more susceptible to HCC than premenopausal women [20]. Median age of patients with HCC was 39 years, which was low in this study. This is similar to other studies conducted in HBV endemic countries in the world [12, 21, 22]. Early age of the acquisition of HBV infection may be the reason for this observation. Differences in oncogenicity of HBV genotypes in sub-Saharan Africa including Ghana together with host genetic and environmental factors such as exposure to dietary aflatoxins could also play a role in the early age of HCC onset in Ghana.

One of the main difficulties in the management of HBV in sub-Saharan African countries including Ghana is HBV DNA testing. HBV viral load is important to determine treatment options; however, this test remains costly and therefore not accessible and affordable for many patients [8]. The cost of antiviral drugs used to treat HBV is also another challenge [12]. In this study, only 22.2% of patients were able to undertake viral load testing, and of those eligible for antiviral therapy, treatment was initiated in only 42.3% of cases. In a retrospective study conducted at a teaching hospital in Ghana, located in Accra, the capital city of Ghana, 43.2% of newly diagnosed HBV patients were able to undertake their viral load [16]. In Ghana, many health centers can screen for viral hepatitis status, but for quantitative determination of viral DNA which is important to determine treatment eligibility and for follow-up of the patients, only few centers have the facilities to perform this test, which is often too expensive for many patients. In the current study site, facilities for quantification of viral DNA were not available in St. Dominic Hospital. Currently, samples for determination of viral load must be taken to a private facility in the capital city and therefore contribute to the high price of the test. In patients with high viral load causing liver inflammation, appropriate antiviral therapy for HBV has been shown to reduce the development of cirrhosis and even reverse it. Furthermore, treatment reduces the risk of hepatocellular carcinoma and improves liver-related and all-cause mortality [7, 23, 24]. Despite the availability of safe and cost-effective drugs (the average cost of generic tenofovir in Ghanaian market is GHS 1,800 (US$128.6) per annum, the cost linked to gross national product is still high in Ghana where most patients pay for their own treatment and nucleoside analogue treatment is usually lifelong [12]. In this study, less than half of the patients who required antiviral treatment were able to initiate therapy on account of cost. HBV diagnostics and antivirals significantly need to be accessible at more affordable prices in Ghana, especially at the level of district hospitals where there is a high burden of HBV compared to urban health facilities. Subsidization of HBV diagnostics and treatment in the National Health Insurance Scheme may improve the care of HBV patients.

In patients with hepatitis B virus, the presence of HBeAg in the serum is an indication of active viral multiplication in hepatocytes and is considered a surrogate marker for the presence of viral DNA [18]. Testing for HBeAg can also identify individuals with a high risk of developing liver cancer and help to determine HBV DNA levels used to decide treatment eligibility [25, 26]. In this study, 4.62% were positive for HBeAg among HBsAg-positive individuals. This reflects a pool of individuals who are highly infectious and serve in supporting viral transmission among Ghanaian population. This was lower in an earlier study of blood donors in Kumasi, Ghana, where HBeAg positivity was 13.3% and the prevalence of HBeAg positivity was 18.0% in a study among HBV patients seeking care at Korle-Bu Teaching Hospital, Ghana [16, 27]. Higher seroprevalence rates of HBeAg-positive infection compared to the current study have been reported in various parts of Nigeria. Forbi et al. [28] reported a prevalence of 19.2% among 572 HBsAg-positive individuals in North Central Nigeria. Moreover, Otegbayo et al. and Lesi et al. reported 10.8% prevalence among blood donors in Ibadan and 11.9% among patients with clinical, ultrasound, and/or histological evidence of liver cirrhosis or HCC in Lagos respectively [29, 30]. Ola et al. [31] in ibadan reported 19.0% of HBeAg prevalence among HBV-positive patients. Sagnelli et al. [32] in Italy also reported a prevalence of 13.6% of HBeAg-negative infections in their study [31]. The typical patient referred to the district hospital in Akwatia was HBeAg negative.

Out of 334 participants, 24.3% (81/334) were pregnant women. Majority of the pregnant women (88.89%) were unable to pay for the viral load test and 32.1% were not able to perform their HBV profile. Mother-to-child transmission (MTCT) of HBV has been found to be a significant mode of hepatitis B transmission. For effective prevention of MTCT, in addition to administering the vaccine and immunoglobulin within 24 hours of delivery, antiviral medication is needed for pregnant women who do not meet treatment criteria but have a high viral load (>200,000 IU/mL) in the third trimester. These women should be treated with antiviral medication (tenofovir) [33]. If measurement of HBV DNA concentrations in pregnant women who are positive for HBsAg is not affordable, the option of starting tenofovir in the third trimester to prevent MTCT will not be possible, and these mothers cannot be referred for further assessment. Even if HBeAg status was used as a surrogate marker of HBV replication, 32.1% may miss effective prevention of MTCT of HBV. In this study, only 1.8% of the pregnant women were positive for HBeAg, which is a sign of low replication and indicates reduced MTCT. Median viral load of pregnant women was 345 which reflects the HBeAg status in the current study. For Ghana to achieve Global Health Sector Strategy to eliminate viral hepatitis B by 2030 with the goal of reducing HBV infection by 90%, especially through MTCT, there should be a conscious effort by policymakers to ensure that all pregnant women who test positive to HBV get access to affordable diagnostics, so that effective preventive measures can be put in place to reduce the risk of MTCT.

The median APRI score among cirrhotic was 3.07, comparable to the recommended cut-off value of 2 and above by WHO as noninvasive diagnosis of cirrhosis among adults in low-income countries [9]. The sensitivity of APRI score with cut-off value of 2 in the diagnosis of liver cirrhosis was 70.2% and specificity was 97.9%. There was improved sensitivity (80.9%) without much loss of specificity (95.5%) when the APRI cut-off value based on the Youden index was reduced to 1.1. The WHO APRI score cut-off of 2 and above performed better than a similar study performed in Ghana among an ambulatory liver cirrhosis patients and other studies performed in Africa [12]. The difference maybe as a result of the stages and causes of liver cirrhosis among the participants of the two studies. Emerging studies from sub-Saharan Africa suggest that the current APRI threshold is too high and should be revised to lower cut-off with rule-in and rule-out criteria to better identify patients with cirrhosis [34, 35]. Further studies should be conducted to determine the appropriate cut-offs for other noninvasive parameters for diagnosing advanced fibrosis and cirrhosis in a patient with CHB in Ghana and sub-Sahara Africa as a whole. Since the majority of our patients could not afford the HBV DNA, one of the key parameters to determine who to treat, depending on APRI score alone, may inadvertently miss patients who may require treatment with antivirals to prevent liver cirrhosis and HCC.

This study had some limitations. Firstly, the diagnosis of liver cirrhosis in this study was based mainly on clinical, laboratory, and radiologic examinations. This method of diagnosis without any histologic basis might have missed patients with early or compensated cirrhosis. Furthermore, screening for anti-HDV antibodies for positive HBV clients is not routinely performed in Ghana because of the lack of resources or laboratory capacity. Consequently, the role of HDV in the progression of cirrhosis and HCC could not be assessed in this study. This data is from a single center, a district hospital, and may not be representative of the general population.

## 5. Conclusion

A relatively high percentage of newly diagnosed CHB patients presented late with liver-related complications. Those who presented with liver cirrhosis and hepatocellular carcinoma were of a younger age group compared to Western counterparts and most were male. Majority of the participants were not able to afford viral load testing and antiviral medication in the case of those who required treatment. Screening of hepatitis B among the general population and inclusion of CHB management in the National Health Insurance Scheme should be encouraged to improve treatment coverage.

## Figures and Tables

**Table 1:** Baseline characteristics of study participants.

Baseline characteristics	Chronic HBV (*n* = 227)	Liver cirrhosis (*n* = 54)	HCC (*n* = 53)	*p* value
Median age (IQR)	32 (27–39)	45 (37–55)	39 (29–46)	0.001
Sex				
Male	80 (35.0)	36 (66.7)	46 (86.8)	0.001
Female	147 (65.0)	18 (33.3)	7 (13.2)	
HBV DNA IU/ML, median (IQR)	1569 (167–4886)	13,816 (2350-117,000)	15,550 (1497-20,666)	0.64
APRI score median (IQR)	0.33 (0.22–0.5)	3.07 (1.39–6.95)	0.92 (0.71-1.12)	0.001
ALT U/L median (IQR)	27.2 (18.2–40)	69 (38–131)	68.9 (49.4-112.8)	0.001
HBeAg^∗^				
Positive	9 (4.62)	0 (0)	2 (3.77)	0.28
Negative	186 (95.38)	53 (0)	51 (96.23)	

^∗^Not all patients could afford HBeAg testing; therefore, some values are not included. Not included are the following: chronic HBV = 32/227, liver cirrhosis = 1/54, and HCC = 2/53.

**Table 2:** Categorization of asymptomatic patients by HBeAg and hepatitis status.

	HBeAg negative (*n* = 175)		HBeAg positive (*n* = 7)	
	Chronic infection	Chronic hepatitis	Chronic infection	Chronic hepatitis
Frequency (*n*, %)	135 (77.1)	40 (22.9)	3 (42.9)	4 (57.1)

ALT reference range 10–40 U/L based on EASL guidelines.

**Table 3:** Summary of characteristics of pregnant women with chronic HBV infection (*n* = 81).

Indicator	Result
Age, median (IQR)	30 (27-34)
Alanine aminotransferase (IU/ML), median (IQR)	19.6 (13.6–29.7)
APRI score, median (IQR)	0.24 (0.18–0.39)
HBV DNA (IU/ML), median (IQR)	345 (10–799)
HBeAg	
No HBeAg test (*n*/*N*, %)	26/81 (32.1)
Proportion positive (*n*/*N*, %)	1/55 (1.8)
Viral load	
No viral load (*n*/*N*, %)	72/81 (88.9)
Proportion with detectable viral load (*n*/*N*, %)	5/9 (55.6)
Proportion with viral load > 200,000 (*n*/*N*, %)	1/9 (11.1)
Treatment	
Proportion requiring treatment (*n*/*N*, %)	2/81 (22.5)
Proportion on treatment (*n*/*N*, %)	1/2 (50.0)

**Table 4:** Predictors of liver cirrhosis and HCC.

Asymptomatic	RRR Std. err. (base outcome)	Std error	*z*	*p* > *z*	95% confidence interval	
*Liver cirrhosis*						
APRI	2.35	0.47	4.30	0.001	1.59	3.48
ALT	1.00	0.01	0.40	0.69	0.99	1.01
Sex	0.48	0.25	-1.44	0.15	0.18	1.31
Age	1.13	0.03	4.66	0.001	1.07	1.19
_cons	0.00	0.00	-5.64	0.001	0.00	0.01
*HCC*						
APRI	1.16	1.05	0.17	0.87	0.20	6.85
ALT	1.00	0.02	0.27	0.79	0.97	1.04
Sex	0.00	0.00	-0.01	0.99	0.00	.
Age	1.24	0.10	2.61	0.001	1.06	1.47
_cons	0.00	0.00	-2.78	0.01	0.00	0.02

APRI = AST-platelet ratio index; AST = aspartate aminotransferase; ALT = alanine aminotransferase.

**Table 5:** Performance of APRI score in the diagnosis of cirrhosis.

APRI cut-off	Sensitivity	Specificity	PPV	NPV
2	70.2	96.8	86.8	91.5
1.1^∗^	80.9	95.5	82.6	94.3

PPV = positive predictive value; NPV = negative predictive value. ^∗^Youden index.

## Data Availability

The data used to support the findings of this study are available from the corresponding author upon request. The corresponding author email is amoakoduah@yahoo.com and his phone number is +233244837310.
